# Olfactory Ensheathing Cells Grafted Into the Retina of RCS Rats Suppress Inflammation by Down-Regulating the JAK/STAT Pathway

**DOI:** 10.3389/fncel.2019.00341

**Published:** 2019-07-25

**Authors:** Jing Xie, Yijian Li, Jiaman Dai, Yan He, Dayu Sun, Chao Dai, Haiwei Xu, Zheng Qin Yin

**Affiliations:** ^1^Southwest Eye Hospital, Southwest Hospital, Third Military Medical University (Army Medical University), Chongqing, China; ^2^Key Laboratory of Visual Damage, Regeneration and Restoration of Chongqing, Chongqing, China

**Keywords:** retinitis pigmentosa, microglia, infiltrated macrophage neuroinflammation, JAK/STAT pathway, olfactory ensheathing cell

## Abstract

The inflammatory microenvironment in the retina plays a vital role in the pathogenesis and progression of retinitis pigmentosa (RP). Microglial inflammatory cytokines production leads to gliosis and apoptosis of retinal neurons, and ultimately, visual loss. Cell-based therapies using grafted olfactory ensheathing cells (OECs) have demonstrated modulation of degenerative microenvironments in the central nervous system (CNS), in a number of animal models. However, mechanisms by which grafted OECs can reduce degeneration in the retina are not well understood. In the present study, we set up an *in vitro* OEC/BV2 microglia co-culture system, and an *in vivo* royal college of surgeons (RCS) rat model, used cell transplantation, immunohistochemistry, RT-PCR, western blot to explore the mechanisms by which OECs affect expression of pro- or anti-inflammatory cytokines and polarization of M(IL-6) and M(Arg1) type microglial activation in the retina. We found that compared with the LPS (Lipopolysaccharide) and olfactory nerve fibroblast (ONF), the OEC and BV2 co-culture group modulate microglial cytokines releasing toward the anti-inflammation, and away from the pro-inflammation, which was followed by higher IL-4 and IL-10 and lower TNF-a and IL-6 in their expression levels. *In vivo*, the transplantation group significantly reduced activated resident microglia/infiltrated macrophage, and expression of pro-inflammatory cytokines in RCS rats retina, increased anti-inflammatory cytokines in transplantation area. Additionally, we found that OECs expressed SOCS3 and down-regulated the JAK2/STAT3 (Janus Kinase 2/Signal Transducer and Activator of Transcription 3) pathway. Thirdly, OEC transplantation reduced Caspase-3 expression, protected inner retinal neurons and photoreceptors and therefore, delayed the visual function degeneration. In conclusion, our data suggest that OECs delay retinal degeneration in RP, at least in part through immunomodulation of microglia via the JAK/STAT pathway.

## Introduction

Retinitis pigmentosa (RP) is a heterogeneous group of inherited retinal degenerative diseases, which lead to photoreceptor cell apoptosis and severe vision loss. Photoreceptor degeneration starts from microglial activation, macrophage infiltration, and accumulation of immunoglobulins and complement, which result in sustained inflammation, macroglia proliferation and progressive apoptosis of retinal neurons (Athanasiou et al., 2018; Ben et al., 2019). Therefore, modulation of microglia activation and inflammatory reaction might be a potential intervention for RP.

In royal college of surgeons (RCS) rats with *Mertk* gene mutation of retinal pigmented epithelium (RPE), microglia become activated, and infiltrate into the outer nuclear layer (ONL), to assist with phagocytosis of photoreceptor debris (Zou et al., 2019). As retinal degeneration continues, blood-retinal barrier (BRB) disruption results in the recruitment of blood-borne macrophages. This is an important step in activating the immune cells and releasing pro-inflammatory cytokines, which amplify the disease process, and leading to photoreceptor apoptosis (Kyger et al., 2013). Studies have already shown that blood-derived immune cells are an important component of the disease-associated microenvironment, and they are considered to be critical mediators of neurodegenerative disease progression, not only in CNS but also in retina (Xu et al., 2009; Sevenich, 2018).

Microglia, resident immune population of the retina, are react to injury as specialized scavengers by promote, and resolve inflammation (Ramirez et al., 2017). In the central nervous system (CNS), resident (microglia) and invading innate immune cells (macrophage) coordinated complex responses to injury, and aiming to restore tissue integrity but can also promote destructive neuroinflammation (Wohleb, 2016; Subramaniam and Federoff, 2017). There are two main microglial/macrophage phenotypes (characterized by morphology, cytokine/chemokine expression, and function). The classically activated phenotype, promotes inflammation by releasing numerous pro-inflammatory cytokines (e.g., IL-6, TNF-α, and MCP), as means of M(IL-6). The alternatively activated phenotype promotes tissue repair and regeneration, by releasing protective/trophic factors (e.g., Arg1, IL-4, and IL-10), and clearing cellular waste debris through phagocytosis, as means of M(Arg1) (Ransohoff, 2016; Edholm et al., 2017). This M(IL-6)/M(Arg1) paradigm has been used to describe the *in vitro* perturbation of macrophages, yet there is evidence that microglia can adopt similar phenotypes, and functions *in vivo* (Hu et al., 2015). Due to these opposing effects of different immune cell phenotypes, recent treatment for neuroinflammation are shifting from complete immune cell suppression to find a balance between M(IL-6)/M(Arg1) phenotypes and searching regulatory molecules that control the two phenotypes polarization switching (Neves et al., 2016).

Several studies in animal models of neurodegenerative disease, including Alzheimer’s disease (Biscaro et al., 2012), Parkinson’s disease (Garrido-Mesa et al., 2013) and RP (Peng et al., 2014), have shown that anti-inflammatory therapies, such as minocycline, have neuroprotective properties. However, the duration of action of minocycline is only 5–10 days, and there is therefore considerable interest in developing a cell-based therapy, which can provide sustained modulation of the inflammatory microenvironment in the degenerative retina (Neves et al., 2016; Zhu et al., 2017).

Olfactory ensheathing cells (OECs) are a unique type of glial cell, which share some features and functions with Schwann cells and astrocytes (Li et al., 1997). OECs have been demonstrated to facilitate glial scar rearrangement, blood vessel formation, axon remyelination, and phagocytosis of cellular debris and pathogens (Chuah et al., 2011; Huo et al., 2012). Microarray analysis of the OEC transcriptome indicates that they express higher levels of a number of innate immune factors, compared to astrocytes and Schwann cells, suggesting an enhanced role in modulating immune cells and neuroinflammation (Vincent et al., 2005). Khankan et al. (2016) suggest that grafted OECs reduce macrophage infiltration, maintain serotonergic (5-HT) axons, and reduce inhibitory chondroitin sulfate proteoglycans (CSPGs) in injured rodent spinal cord, leading to the restoration of motor function. Zhang et al. (2017) have shown that OEC transplantation reduces inflammatory cell infiltration in spinal cord injury, and promotes a shift in the macrophage phenotype from M(INF-γ) to M(IL-4). Although microglial phenotype switching is not fully understood, there is thought to be an important role for the JAK/STAT (janus kinase/signal transducer and activator of transcription) pathway and some binding proteins, which may accelerate the pace of immune cell suppression, and toward rebalance M(IL-6)/M(Arg1) activity (Hu et al., 2015). In retina, some studies have reported a dynamic shift in microglia changing profile of recognized M(IL-6)- to M(Arg1)- following acute light damage (Jiao et al., 2015). In rd1 mice, the microglia orchestrate a continuous spectrum which is activated and polarized to a M(IL-6) phenotype during acute retinal degeneration (Zhou et al., 2017). Recent work has shown that the JAK/STAT pathway is central to the determination of M(IL-6) vs. M(Arg1) microglial subtypes (Sica and Bronte, 2007). Upon binding to JAK, members of the IL-6 family of cytokines activate the JAK/STAT signaling pathway (Tam and Ma, 2014; Hu et al., 2015; Qin et al., 2016). It has also been demonstrated that transplanted OECs promote neurological functional recovery in traumatic brain-injured rats via the JAK/STAT3 pathway (Fu et al., 2015).

Our previous research has found that there is microglial activation in the degenerative period of RCS rat retina (Liu et al., 2013; Li et al., 2016). However, as BRB breakdown and macrophages infiltrate into the retina during retinal degeneration (Shen et al., 2010; de Hoz et al., 2016), neither the polarization of resident microglia, and its effect in RCS rat, nor the ways to regulate this polarization, has ever been investigated.

In this study, we investigated the effect of OECs on activated microglia in an *in vitro* co-culture system. Co-culture of OECs with BV2 cells (an immortalized microglial cell line) reduced lipopolysaccharide (LPS)-induced microglial activation and produced microglial polarization toward the M(Arg1) phenotype. Immunofluorescent staining showed OECs were SOCS3 positive. These microglial modulation effects of OECs may mediated by downregulation of the JAK2/STAT3 signaling pathway. We also used a rodent model of inherited retinal inflammation and degeneration (RCS rat) to study the *in vivo* effect of OEC implantation into the retina, during the chronic stages of the disease process. Immunohistochemical data, and gene and protein quantification, demonstrated that OEC transplantation delayed the degeneration of retinal neurons and photoreceptors via an anti-apoptotic mechanism, inhibited microglial/macrophage activation, and reduced pro-inflammation cytokines, by down-regulating the JAK2/STAT3 pathway. These results further confirm that microglial activation and a pro-inflammatory environment play a pivotal role in the retinal degeneration of RCS rats, and that OEC transplantation can preserve visual function partly through regulating the microglia-mediated inflammatory environment.

## Materials and Methods

### Animal Models and Ethical Approval

We used black-eyed RCS rats as a model of retinal dystrophy, and “rdy” rats as non-retinal dystrophic controls; both supplied by the Animal Centre of the Third Military Medical University (TMMU). Rats were maintained in the animal facility of the Southwest Eye Hospital, the TMMU. Housing rooms had regular day and night light-cycles (12:12 h). All rats were sacrificed using a carbon dioxide inhalation chamber. All surgical procedures and post-operative care were conducted in accordance with protocols approved by the TMMU Institutional Animal Care and Use Committee.

### Chemicals and Reagents

Fetal bovine serum (FBS), Dulbecco’s modified Eagle’s medium/Ham’s Nutrient Mixture (DMEM/F12 plus GlutaMAX), Hank’s balanced salt solution (HBSS), phosphate buffer (PBS), and other cell culture reagents were obtained from Thermo-Fisher Corporation (Beijing, China). Trypsin and penicillin/streptomycin solutions were obtained from Hyclone (Bejing, China). Lipopolysaccharide (LPS), 2-cyano-3 (3,4-dihydroxyphenyl)N-(benzyl)2-propenamide (Tyrphostin AG490), poly-L-lysine (PLL), and pentobarbital sodium were purchased from Sigma-Aldrich (Shanghai, China). Glyceraldehyde-3-phosphate dehydrogenase (GAPDH) and primers were purchased from Thermo Fisher Scientific. The primary and secondary antibodies used for immunohistochemistry and WB are listed in Table 1.

**TABLE 1:** Primary antibodies used.

**Antibody**	**Manufacturer’s catalog or lot number**	**Dilution**
Mouse anti-NGFRp75	Santa Cruz, sc-271708	1: 50
Rabbit anti-Iba1	Wako, 019-19741(IFC) 016-20001(WB)	1: 500 1:1000
Rabbit anti-TMEM119	Abcam, ab185333 (IHC) Santa Cruz, sc-244341 (WB)	1: 50 1: 500
Mouse anti-PKCα	Santa Cruz, Santa Cruz, sc-8393	1: 500
Mouse anti-Rhodopsin	Abcam, ab5417	1: 1000
Rabbit anti-S-100β	Abcam, ab868	1: 200
Rabbit anti-Caspase-3 Mouse anti-β-actin Rabbit anti-JAK2 Rabbit anti-STAT3 Rabbit anti-SOCS3 Rabbit anti-pJAK2 Rabbit anti-pSTAT3	Abcam, ab13847 Cell signal technology, 3700 Cell signal technology, 3230 Cell signal technology, 12640 Abcam, ab16030 Cell signal technology, 3776 Cell signal technology, 9145	1: 200 1:2000 1: 1000 1: 1000 1:100 1: 1000 1: 1000

### Primary OEC and ONF Culture and Purification

The olfactory bulbs of adult control rats were used to harvest OECs and olfactory nerve fibroblasts (ONFs, for use as a negative control), and we used differential cell adhesiveness to purify OECs and ONFs (Huo et al., 2011; Dai et al., 2012; Xie et al., 2017). Briefly, olfactory bulbs were removed and put into a 10 mm dish. After carefully isolated of the pia mater and vascular membrane, the olfactory glomerular layers and nerve layer were separated. The nerve layer was cut into 0.5 mm^3^ pieces, placed in 0.125% trypsin to digest for 15 min at 37°C. The cells were cultured with DMEM/F-12 containing 10% FBS and 1% penicillin/streptomycin and inoculated into a six-well plate coated with PLL. Culture medium were first changed for the 5th day, and then changed every 3 days until 2 weeks. After several cycles of differential cell adhesiveness, OECs, and ONFs were easily separated. OECs or ONFs were dissociated into suspension and then labeled with lentiviruses carrying the enhanced green fluorescent protein (LV-EGFP) prior to subretinal transplantation. The production, purification and infection of LV-EGFP was according to previous reports (Xie et al., 2017).

### Culture of BV2 Microglia

BV2 murine microglial cell line was given by Dr. Guo from the Neurological Surgery Department of Southwest Hospital. Cells were seeded into six-well plates at a concentration of 10^5^ cells/well in DMEM containing 10% FBS and 1% penicillin/streptomycin, as previously described (Li et al., 2016).

### Co-culture of OEC or ONF Cells With BV2 Microglia Cells

**FIGURE 1:**
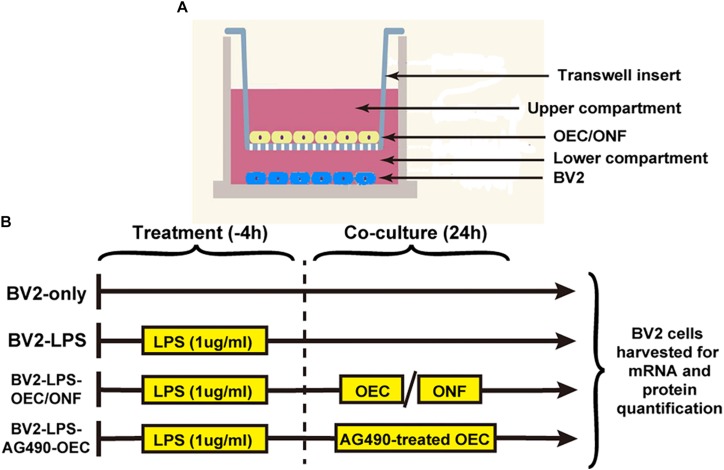
Design of the *in vitro* co-culture experiment. **(A)** Schematic illustrating the co-culture system, with a Transwell insert hanging in each well of a six-well plate. **(B)** Experimental schedule. AG490-OECs are OECs that have been pre-treated for 1 h with AG490 (a JAK2 antagonist) prior to co-culture.

The co-culture experimental design is shown in Figure 1. We maintained the BV2 mouse microglia cell line in six-well plates (10,000 cells each). To induce BV2 cell reactivity we used 1 μg/ml of LPS diluted in basal medium for 4 h. OECs or ONFs were seeded into Transwell plate inserts (Millipore) (10,000 cells each). BV2 cultures were co-cultured with the OECs for 24 h (or ONFs as a negative control) to explore their role in immune modulation. To study the role of the JAK/STAT signaling pathway in OECs, the JAK2 antagonist Tyrphostin AG490 (50 μm) (Ito et al., 2010; Hou et al., 2017) was added to the OEC culture 1 h before co-culture with BV2 cells, in relevant experiments.

### Subretinal Injection

After purification, OECs or ONFs were separately detached and implanted into the subretinal space of P30d RCS rats, as previously described (Xie et al., 2017). Briefly, we exposed the temporal part of the sclera, and a 30G needle was used to reach the subretinal space by incision of the sclera, choroid, and retinal pigment epithelium. A Hamilton syringe with a 33G needle was attached to the choroid layer and slowly injected 10^5^ cells (in 3 μl PBS) into the subretinal space of the right eye. The contralateral eye was injected 3 μl PBS with the same surgical procedures. When the fundus was observed, we could see a white flat detachment of the retina at the transplantation site.

### Electroretinogram (ERG)

Electroretinogram recordings were performed as previously described (Dai et al., 2017; Xie et al., 2017). We dark-adapted rats for 12 h, and prepared under dim red light. Compound tropicamide eye drops were used to dilate pupils. Before recording, artificial tears was applied to allow the platinum recording electrode contact with the cornea and also prevent dehydration. The needle electrode was fixed under the skin of both sides of the rat’s nasal side as a reference electrode, while the ground electrode was placed under the tail. The signal amplifier bandwidth was 0.1–300 Hz, without notch filtering. “RETI-port” software was used to acquire and control stimulus delivery data, running on Roland Electrophysiological Systems hardware (Brandenburg, Germany). The amplitude of the a-wave of ERG was calculated from the baseline to the first trough, and the amplitude of the b-wave was from the trough of the a-wave to the first peak. To improve the signal-to-noise ratio, the inter-stimuli-intervals were longer than 30 s.

### Tissue Sample Preparation

For immunofluorescence staining, we firstly intramuscular injection of pentobarbital sodium (10 mg/kg), and then used normal saline and 4% paraformaldehyde (PFA) to transcardially perfused rats. After taking out the eyeball, the cornea, iris, lens were removed, and the eyecups were put in 4% PFA at 4°C for 0.5 h, then dehydrated in a 30% sucrose solution at 4°C overnight. Sections (10 μm thick) were cut using a freezing microtome (Leica, Germany) at −20°C, then air-dried overnight at 25°C, and stored at −20°C for further immunofluorescence staining. For WB and PCR analysis, rats were transcardially perfused with normal saline and retinas were rapidly removed, immediately frozen in liquid nitrogen, and stored at −80°C for further study.

### Immunofluorescence Staining

Olfactory ensheathing cells and BV2 cells were plated on PLL-treated cover slips. Following each treatment, cells were fixed in 4% PFA for 10 min and rinsed with PBS for three times. Cover slips and sections were permeabilized with 0.03% Triton X-100 for 10 min, and then blocked with 1% goat serum for 0.5 h at 37°C. Cover slips and sections were incubated in primary antibodies at 4°C overnight (Table 1 showed primary antibody dilutions). After washing off the primary antibody with PBS, secondary antibodies (1:1000; Invitrogen, United States) were applied at 37°C in the dark for 0.5 h. 4′,6-diamidino-2-phenylindole (DAPI) (Beyotime, China) was used to counterstain nuclei for 10 min in the dark at room temperature. Sections were washed in PBS for three times, and mounted using anti-fade mounting medium (Beyotime, China). Leica SP5 microscope was used to take confocal micrograps at the Central Laboratory in TMMU (Leica Microsystems, Wetzlar, Germany).

### RNA Extraction and Real-Time PCR

Real-time polymerase chain reaction (RT-PCR) was implemented as previously described (Fu et al., 2017; He et al., 2017). Briefly, Trizol reagent (Sigma-Aldrich) was used to extract total RNA according to the manufacturer’s instructions, and then reverse transcribed with an oligo (dT) primer. Sybr Primix EX TaqTM II and a Takara Thermal Cycler Dice^TM^ Real Time System (Takara Bio Inc. Kusatsu, Shiga, Japan) was used to perform Real-time PCR amplification, according to the manufacturer’s protocols. The selected primers are listed in Tables 2, 3. All the data were normalized to GAPDH expression. The experimental groups were then normalized to control groups expression.

**TABLE 2:** PCR primer sequences (rat) used to detect pro- and anti-inflammatory cytokines in RCS rats’ retina.

**Genes (rat)**	**Forward primer**	**Reverse primer**
GAPDH	AAGGTCGGTGTGAACGGATT	TGAACTTGCCGTGGGTAGAG
TNF-α	CTCAAGCCCTGGTATGAGCC	GGCTGGGTAGAGAACGGATG
IL-6	TCCTACCCCAACTTCCAATGC	TAGCACACTAGGTTTGCCGAG
MCP-1	GCTGTAGTATTTGTCACCAA GCTCAA	GTACTTCTGGACCCATTCCTT ATTG
ICAM-1	AGTGCTGTACCATGATCAGA ATACCT	TAAATGGACGCCACGATCAC
Arg1	CCTGAAGGAACTGAAAGG AAAGTT	GCAAGCCGAT GTACACGATGT
IL-4	ACCCTGTTCTGCTTTCTC	GTTCTCCGTGGTGTTCCT
IL-13	AATCCCTGACCAACATCT	ATAAACTGGGTACTTCG
Iba1	CGAATGCTGGAGAAACTTGG	GTTGGCTTCTGGTGTTCTTTG
TMEM119	GCTACGCTTTCTTCACGTTGC	AACCAATCAGGAAGTGGGGT
PKC-α	TTTCTTCCCCCACCCAATCC	AGGGTCCAAGTCTCTTTGTTTCC
Rhodopsin SOCS3	AACCTTGAGGGCTTCTTTGCCA TTCTTTACCACCGACGGAAC	AAGTTGCTCATGGGCTTGGAGA CACGTTGGAGGAGAGAGGTC

**TABLE 3:** PCR primer sequences (mice) used to detect pro- and anti-inflammatory cytokines in BV2 cells.

**Genes (mice)**	**Forward primer**	**Reverse primer**
GAPDH	CAGCAACTCCCACTCTTCCAC	TGGTCCAGGGTTTCTTACTC
TNF-α	TGTGCTCAGAGCTTTCAACAA	CTTGATGGTGGTGCATGAGA
IL-6	TAGTCCTTCCTACCCCAATTTCC	TTGGTCCTTAGCCACTCCTTC
Arg1	ACAAGACAGGGCTCCTTTCAG	GGCTTATGGTTACCCTCCCG
IL-4	ATCCATTTGCATGATGCTCT	GAGCTGCAGAGACTCTTTCG
Iba1	GGATTTGCAGGGAGGAAAAG	TGGGATCATCGAGGAATTG
TMEM119	GTGTCTAACAGGCCCCAGAA	AGCCACGTGGTATCAAGGAG

### Western Blot

Samples (either BV2 cells or rat retinas) were collected to a glass homogenizer and grinded with radioimmunoprecipitation assay (RIPA) buffer containing protease inhibitor (Beyotime), and then incubated on ice for 15 min. The supernatant was isolated by supercentrifuge at 15,000 *g*/min for 5 min at 4°C. The total protein concentration in each sample was quantified using the bicinchoninic acid (BCA) Protein Quantitation Kit (Beyotime). The protein samples were added with 5× SDS (Sodium Dodecyl Sulfate) loading buffer (Beyotime) in a 4:1 ratio and then subjected to SDS-PAGE (Polyacrylamide Gel Electrophoresis; Beyotime). After electroblotting transferred onto polyvinylidene fluoride (PVDF) membranes (Bio-Rad), the 5% non-fat milk in tris-buffered saline Tween (TBST) was used to block and different primary antibodies were incubated with at 4°C overnight (Table 1). Washed the membranes with TBST three times and incubated with horseradish peroxidase-conjugated sheep anti-mouse (1:3000; Santa Cruz Biotechnology) or goat anti-rabbit immunoglobulin-G (1:3000; Santa Cruz Biotechnology) as secondary antibodies for 2 h at 25°C. After washing three times with TBST, chemiluminescence detection reagents were used to visualize the bands on the membranes. β-actin was used as a internal control.

### Statistical Analysis

All data are expressed as mean ± standard deviation (SD). Data were analyzed using SPSS software (v21, IBM, Armonk, NY, United States). The data were evaluated using unpaired two-sample *t*-tests, to compare the means at the same time points between the experimental and control groups; one-sample *t*-tests where test distributions were compared to a specific value, or two-way ANOVA followed by Fisher’s protected least-significant difference *post hoc* tests. Values of *p* < 0.05 were considered statistically significant.

## Results

### Resident Microglia Activation and Macrophages Infiltration During Retinal Degeneration

**FIGURE 2:**
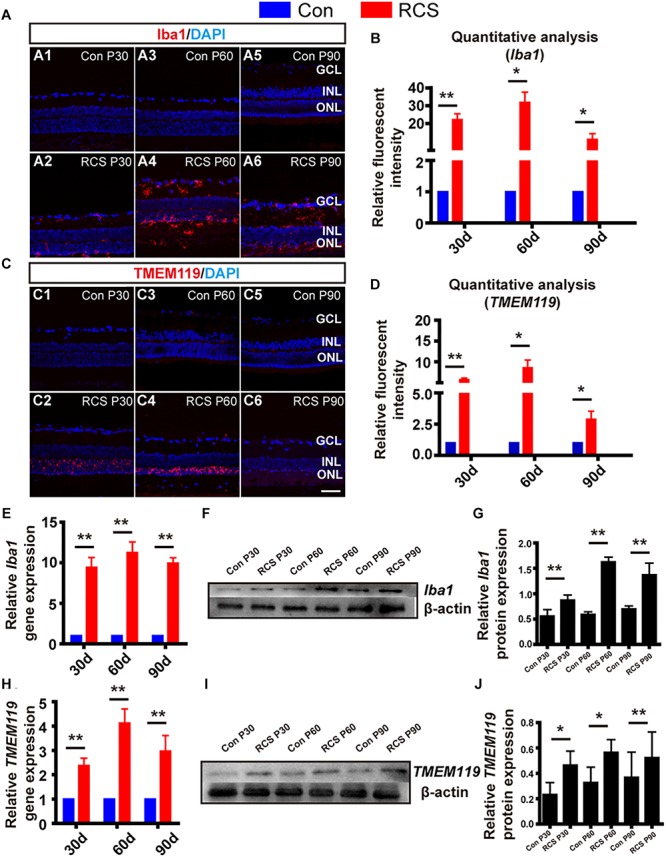
Distribution and activation marker change of resident microglia and infiltrated macrophage in RCS rats. **(A)** Immunohistological images, labeled with Iba1 (red), and DAPI (blue) in retinal slices from control (rdy) rats **(A1,A3,A5)**, and RCS rats of different post-natal ages **(A2,A4,A6)**. **(B)** Quantitative group analysis of relative Iba1 fluorescence intensity at different ages, normalized to each control (*n* = 3 per bar). **(C,D)** Same as **(A,B)**, but for TMEM119 (red) **(E)** mRNA expression level of Iba1 in RCS retinas, relative to expression in control retinas of the same age (*n* = 3 per bar). **(F)** Example western blot of Iba1 protein levels. **(G)** Quantitative group data of Iba1 protein levels in RCS retinas, normalized to β-actin expression levels, and compared to each control retinas (*n* = 3 per bar). **(H–J)** Same as **(E–G)** but for TMEM119. ^*^p < 0.05, ^∗∗^*p* < 0.01; scale bars: 50 μm.

In order to investigate the dynamic change of resident microglia activation and infiltrated macrophages during retinal degeneration, we performed immunohistochemistry on retinal sections from different age of RCS and normal control rats. We stained ionized calcium-binding adaptor molecule-1 (Iba1), a marker for both activated microglia and macrophages (Li et al., 2016), and used TMEM119 to specifically label activated resident microglia (Bennett et al., 2016). In retinas of normal control rats, we rarely saw staining with either Iba1 or TMEM119 (Figures 2A1,A3,A5,C1,C3,C5). In contrast, in RCS rats we saw Iba1-positive and TMEM119-positive cells, whose numbers varied during the course of retinal degeneration, over the first 90 post-natal days.

During early-stage degeneration (P30), Iba1-positive cells in RCS rat retinas were localized in the ganglion cell layer (GCL) and ONL (Figure 2A2). There was large number of TMEM119-positive cells localized in the ONL only (Figure 2C2). At mid-stage (P60), there was an increased number of Iba1-positive cells, and these were found across the entire retina. These cells had a ramified appearance, suggesting migrating, activated microglia/macrophages (Figure 2A4). TMEM119-positive cells remained localized to the ONL but their immunofluorescence became more intense (Figure 2C4). At late-stage (P90), there was obvious apoptosis of retinal cells, and the number of both Iba1-positive and TMEM119-positive cells decreased, compared to P60 (Figures 2A6,C6).

Secondly, we performed quantitative analysis of relative fluorescence intensity, compared to control retinas. This showed that both Iba1 and TMEM119 intensity was significantly elevated at P30 (Iba1: 22.14 ± 3.3 fold; TMEM119: P30: 5.71 ± 0.42 fold; *n* = 3 per group; *p* < 0.01 for each vs. controls). Peak expression was at P60 (Iba1: 31.79 ± 5.78 fold; TMEM119: 8.59 ± 1.86 fold; *n* = 3 per group; *p* < 0.01 for each vs. control). Expression then fell at P90, but was still significantly elevated (Iba1: 11.06 ± 3.26-fold; TMEM119: 2.88 ± 0.65-fold *n* = 3 per bar; *p* < 0.01 for each vs. controls) (Figures 2B,D).

Thirdly, to investigate changes in Iba1 and TMEM119 at the mRNA and protein level, we also performed RT-PCR and WB. Compared to control rats of the same age, the mRNA expression of Iba1 was increased in RCS retinas by more than ninefold at all ages (*p* < 0.01 for comparisons to control at all ages, *n* = 3 per group; Figure 2E). The expression of TMEM119 was increased by more than twofold at all ages (*p* < 0.01 for comparisons to control at all ages, *n* = 3 per group) (Figure 2H). In WB analysis (e.g., Figures 2F,I), the densities of Iba1 and TMEM119 bands were significantly higher in the RCS rats compared with the same age of control rats (Iba1: Con vs. RCS: P30: 0.87 ± 0.1 vs. 0.56 ± 0.13; P60: 0.59 ± 0.05 vs. 1.63 ± 0.09; P90: 0.7 ± 0.06 vs. 1.38 ± 0.23 *n* = 3 per group; *p* < 0.05; Figure 2G) (TMEM119: Con vs. RCS: P30: 0.23 ± 0.09 vs. 0.46 ± 0.11; P60: 0.33 ± 0.12 vs. 0.57 ± 0.1; P90: 0.37 ± 0.2 vs. 0.52 ± 0.2 *n* = 3 per group; *p* < 0.05; Figure 2J).

### Retinal Degeneration Induces JAK2/STAT3 Pathway Activation and Downstream Cytokines Expression

**FIGURE 3:**
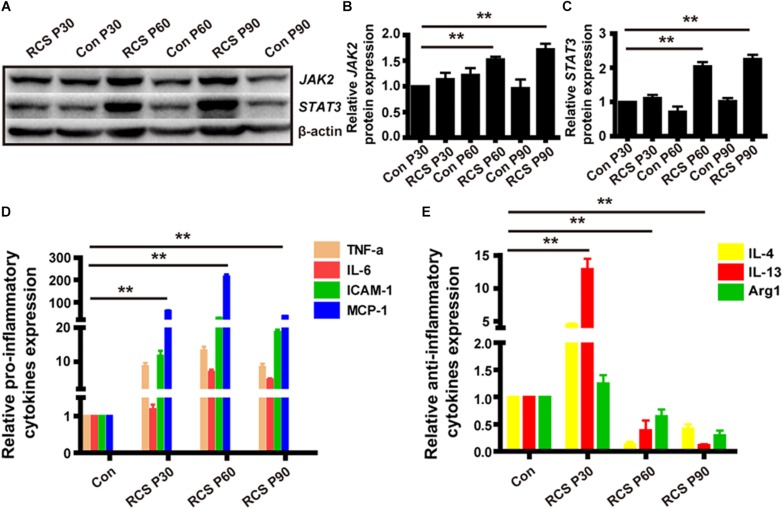
JAK2/STAT3 activation and the change of downstream cytokines expression in RCS rat retinas. **(A)** WB results for protein levels of JAK2 and STAT3 in retinas from RCS rats and control rats of different ages. **(B)** Group data showing JAK2 expression by western blot in RCS rats. **(C)** The same as **(B)**, but for STAT3. **(D)** mRNA expression levels of pro-inflammatory cytokines: TNF-α, IL-6, ICAM-1, and MCP-1 (n = 3 per bar). **(E)** mRNA expression levels of anti-inflammatory cytokines: IL-4, IL-13, and Arg1 (n = 3 per bar). ^*^p < 0.05, ^∗∗^*p* < 0.01.

We next examined the expression of pJAK2, pSTAT3, JAK2 and STAT3 because the JAK/STAT pathway is a well-known modulator of microglia activation and pro-inflammatory cytokines expression. From WB result of retinal tissues from different age of RCS and normal control rats, we can find the expressions of pJAK2, pSTAT3, JAK2, and STAT3 were all up-regulated during retinal degeneration in RCS rats (*n* = 3 per group, Supplementary Figure S1) (JAK2: P30: 1.11 ± 0.09 fold; P60: 2.04 ± 0.13 fold; P90: 2.26 ± 0.12 fold; *n* = 3 per group; *p* < 0.01 for P60 and P90 RCS vs. control; STAT3: P30: 1.13 ± 0.12-fold; P60: 1.53 ± 0.05-fold; P90: 1.72 ± 0.11-fold; *n* = 3 per group; *p* < 0.01 for P60 and P90 RCS vs. control) (Figures 3A–C). We next examined whether retinal degeneration induced expression of classically identified STAT inducible genes. The results shown in Figure 3D reveal that retinal degeneration induced the high expression of pro-inflammatory factors such as TNF-α, IL-6, ICAM-1, and MCP-1 in a time-dependent manner, which is indicative that microglia/macrophages were activated with “classically” phenotype (Qin et al., 2016). In contrast, expression of anti-inflammatory factors such as Arg1, IL-4, and IL-10 tended to be more highly expressed at P30 and were significantly reduced at P60 and P90 (*n* = 3 at each time-point; *p* < 0.01 for each vs. controls, Figure 3E). These results collectively demonstrate that retinal degeneration induces activation of JAK2 and STAT3 and downstream gene expression in microglia/macrophages indicative of the pro-inflammatory phenotype. Alternatively activation, responsible for anti-inflammatory effects, are increased in early-stage degeneration (but at much lower multiples of the control level than pro-inflammatory cytokines). Anti-inflammatory cytokine gene expression is then reduced from P60, and this reduction in anti-inflammatory mediators may also have a pro-inflammatory outcome.

### OECs Inhibit LPS Induced BV2 Microglia Activation, and Changed the Expression Level of Inflammatory Factors and JAK2/STAT3 Pathway *in vitro*

To examine the immunomodulating potential of OECs against inflammation *in vitro*, LPS-induced microglia activation were established in BV2 cell line (experimental design in Figure 1). BV2 microglial cells were treated with LPS (1 μg/mL) for 4 h to release the pro-inflammatory factors (Dai et al., 2011; Li et al., 2016). We then co-cultured the BV2 cells with either OECs, or ONFs (control), for another 24 h, before collecting the BV2 for further use.

**FIGURE 4:**
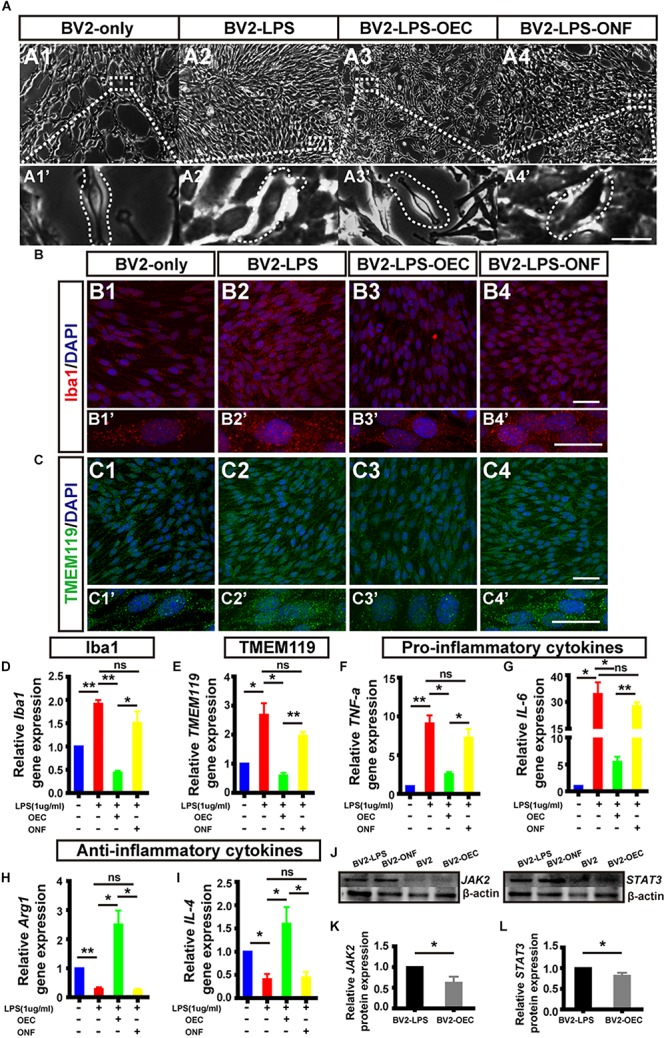
OECs inhibited microglial activation *in vitro* and changed the expression of pro- and anti-inflammatory factors. **(A1)** Optical microscopy image showing density of normal cultured BV2 cells (BV2-only). Lower panel **(A1’)** shows an enlargement of the area marked, which shows morphology of microglia. **(A2)** The same for BV2 cells treated with LPS for 4 h (BV2-LPS). **(A3)** The same for BV2 cells treated with LPS for 4 h and co-cultured with OECs (BV2-LPS-OEC). **(A4)** The same for BV2 cells treated with LPS for 4 h and co-cultured with ONFs (BV2-LPS-ONF). Scale bars: 100 μm. in upper panels, 20 μm in lower panels **(B1–B4)** Immunohistochemistry images showing staining with Iba1 (red) and DAPI (blue) for the same groups as in **A1–A4**. Scale bars: 50 μm. **(B1’–B4’)** Lower panel shows an enlargement of the cells in **B1–B4**. Scale bars: 20 μm. **(C1–C4)** The same for staining with TMEM119. Scale bars: 50 μm. **(C1’–C4’)** Lower panel shows an enlargement of the cells in **C1–C4**. Scale bars: 20 μm. **(D)** mRNA expression level of Iba1 after 24 h co-culture. **(E)** TMEM119 mRNA expression. **(F,G)** Expression of pro-inflammatory cytokines: TNF-α and IL-6, respectively. **(H,I)** Expression of anti-inflammatory cytokines: Arg1 and IL-4, respectively. **(J)** Example WB of JAK2 and STAT3, with β-actin as loading control. **(K)** Quantified JAK2 protein expression by WB (*n* = 3 per group). **(L)** Same for STAT3 (*n* = 3 per group). ^*^p < 0.05, ^∗∗^*p* < 0.01.

**FIGURE 5:**
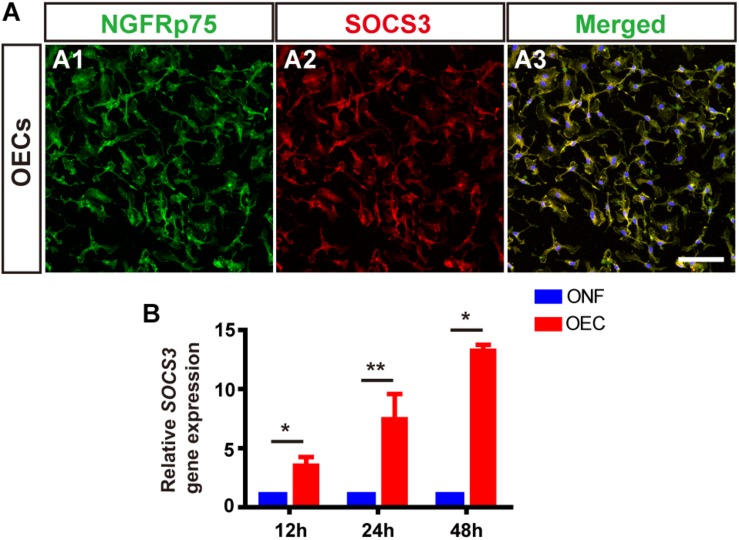
OECs express SOCS3 *in vitro*. **(A)** Immunohistology images, labeled for NGFRp75 (*green*), SOCS3 (*red*) and DAPI (*blue*) in the purified OECs. Scale bar: 50 μm. **(B)** mRNA expression level of SOCS3 after 12, 24, and 48 h co-culture with LPS-BV2 cells compared with ONFs (*n* = 3 per group). ^*^p < 0.05, ^∗∗^*p* < 0.01.

Following LPS treatment, we saw increased cell density consistent with microgliosis (Figures 4A1 vs. A2). BV2 cells in the untreated (BV2-only) group showed round/oval or ramified morphology (Figure 4A1’). After stimulation with LPS, the morphology of BV2 cells changed into an amoeboid shape (Figure 4A2’). BV2 cells in the group co-cultured with OECs (BV2-LPS-OEC) showed similar morphology to the BV2-only group (Figures 4A3,A3’), whereas BV2 cells co-cultured with ONFs (control) showed similar morphology to the BV2-LPS group (Figures 4A4,A4’). Immunocytochemistry using the microglial marker Iba1 (Figures 4B1–B4’) and TMEM119 (Figures 4C1–C4’) demonstrated increased expression in the LPS-stimulated BV2 group and BV2-LPS-ONF group, compared to the BV2-only, and BV2-LPS-OEC group. BV2 microglial cells were treated with LPS for 4 h to induce the pro-inflammation phenotype. The mRNA expression of microglial markers (Iba1, TMEM119, Figures 4D,E) and pro-inflammation cytokines (TNF-α, IL-6, Figures 4F,G) in BV2 cells was enhanced significantly, while the anti-inflammation cytokines (Arg1, IL-4, Figures 4H,I) decreased greatly. These trends were markedly reversed after co-culture with OECs. Thirdly, the results from WB analysis revealed that the JAK2 and STAT3 expressions in LPS-stimulated BV2 cells co-cultured with OECs were significantly lower than those in the LPS-stimulated BV2 group (JAK2: 0.62 ± 0.15-fold in BV2-LPS-OEC group; STAT3: 0.81 ± 0.07-fold in BV2-LPS-OEC group; compared with the BV2-LPS group, *p* < 0.05) (Figures 4J–L). Moreover, we found purified OECs were immunopositive for the specific NGFRp75 (Figure 5A1) and SOCS3 (Figure 5A2) using double immunofluorescence staining (Figure 5A3). The mRNA expression of SOCS3 in OECs was enhanced significantly from 12 to 48 h after co-cultured with LPS induced BV2 cells, ONFs were used as negative control [SOCS3: 12 h: 3.5 ± 0.74 fold; 24 h: 7.45 ± 2.12 fold; 48 h: 13.24 ± 0.51 fold; *n* = 3 per group; *p* < 0.05 for 12 and 48 h OECs vs. ONFs; *p* < 0.01 for 24 h OECs vs. ONFs) (Figure 5B)]. These results suggested that OECs could inhibit microglia activation, reduce expression of pro-inflammatory cytokines (TNF-α, and IL-6), increase the expression of anti-inflammatory cytokines (Arg1 and IL-4), down-regulate JAK2/STAT3 pathway by over-expressed SOCS3 *in vitro*.

### OECs Regulate the Expression Level of Pro- and Anti-inflammatory Factors of Microglia in a JAK2/STAT3-Dependent Manner

**FIGURE 6:**
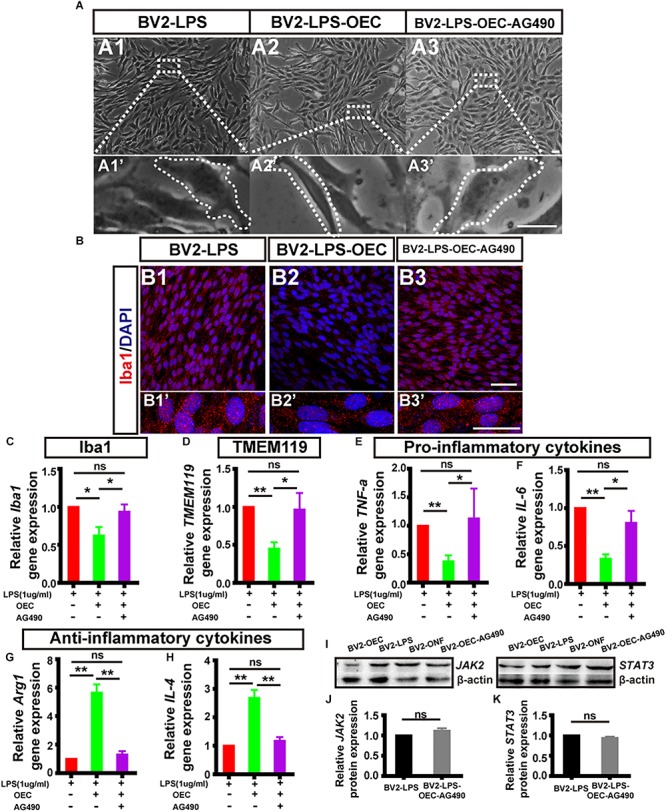
The expression level of pro- and anti-inflammatory factors shift in activated microglia induced by OECs. **(A1)** Optical microscopy image showing density of BV2 cells treated with LPS for 4 h (BV2-LPS). Lower panel **(A1’)** shows an enlargement of the area marked which shows the morphology of microglia. **(A2)** The same for BV2 cells treated with LPS for 4 h and co-cultured with OECs (BV2-LPS-OEC). **(A3)** The same for BV2 cells treated with LPS for 4 h and co-cultured with OECs pre-treated with AG490 (BV2-LPS-OEC-AG490). Scale bars: 100 μm in upper panels, 20 μm in lower panels **(B1–B3)** Immunohistochemistry images showing staining with Iba1 (red) and DAPI (blue) for the same groups as in **A1–A3**. Scale bars: 50 μm. **(B1’–B3’)** Lower panel shows an enlargement of the cells in **B1–B3**. Scale bars: 20 μm. **(C)** mRNA expression level of Iba1 after 24 h co-culture. **(D)** TMEM119 mRNA expression. **(E,F)** Expression of pro-inflammatory factors: TNF-α and IL-6, respectively. **(G,H)** Expression of anti-inflammatory factors: Arg1 and IL-4, respectively. **(I)** Example WB of JAK2 and STAT3, with β-actin as loading control. **(J)** Quantified JAK2 protein expression by WB (*n* = 3 per group). **(K)** Same for STAT3 (*n* = 3 per group). ^*^p < 0.05, ^∗∗^*p* < 0.01.

To determine if JAK2/STAT3 pathway activation was causally involved in the OEC-induced change of pro- and anti-inflammatory factors in microglia, we incubated OECs with AG490, a specific chemical antagonist of JAK2 (at 50 μM, for 1 h) followed by co-culture with LPS-stimulated BV2 for 24 h. We found that the morphology of BV2 co-cultured with AG490-pretreated OECs was amoeboid (Figure 6A3), similar to the LPS-induced BV2 group (Figure 6A), unlike the morphology of BV2 cells co-cultured with OECs (Figures 6A2,A2’). Additionally, immunocytochemistry showed increased expression of Iba1 in the BV2-LPS-OEC-AG490 group (Figure 6B).

Secondly, we measured the mRNA expression levels of microglial activation markers (Iba1, TMEM119), pro-inflammatory factors (TNF-α, IL-6), and anti-inflammatory factors (Arg1 and IL-4) of BV2 in each group, using RT-PCR. We found that AG490-pretreatment reversed the changes in mRNA expression by BV2 cells associated with OEC co-culture, such that the BV2-LPS-AG490-OEC group was statistically indistinguishable from the BV2-LPS group, and significantly different from the group treated with normal OECs (Figures 6C–H).

Finally, we studied the expression of JAK2/STAT3 proteins in BV2 cells co-cultured with AG490-pretreated OECs, using WB. We found that expression of pJAK2, pSTAT3, JAK2, and STAT3 was very similar between the BV2-LPS, BV2-LPS-ONF groups and the BV2-LPS-OEC-AG490 groups (*n* = 3 per group, Supplementary Figure S2) (JAK2: 1.12 ± 0.59-fold in BV2-LPS-OEC-AG490 group; STAT3: 0.94 ± 0.03-fold in BV2-LPS-OEC-AG490 group; compared with the BV2-LPS group, *p* > 0.05) (Figures 6I–K). These results collectively demonstrated that pretreatment of OECs with AG490 blocked their effects on pro/anti-inflammatory cytokines expression, suggesting that this effect of OECs is dependent on the JAK2/STAT3 pathway in activated BV2 microglia cells.

### OECs Reduce the Classical Immune Cell Activation and the Expression Levels of Pro-inflammatory Factors *in vivo*

In order to understand whether OECs can show similar effect on activated microglia/macrophages *in vivo*, and whether they can modulate the pro-inflammatory microenvironment, we assessed the properties of resident microglia, and infiltrated macrophages, and the level of inflammatory markers, 4 weeks after subretinal OEC transplantation in live RCS rats. To do this, we successfully produced EGFP-labeled OECs, using transfection via a lentiviral vector, and these labeled cells were injected subretinally (Supplementary Figure S3). Rats were then sacrificed at 4 weeks post-transplantation, and their retinas isolated for further study.

**FIGURE 7:**
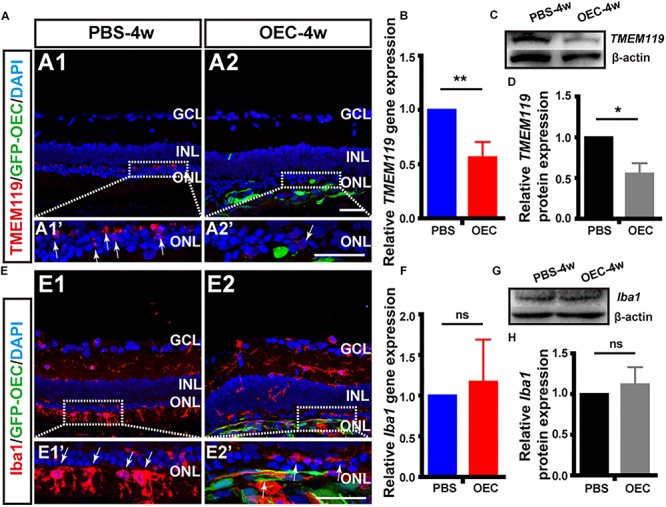
OECs reduce the classical microglial activation *in vivo*. **(A1)** Immunohistology image of a retina from a RCS rat, 4 weeks following subretinal injection of PBS, labeled with Iba1 (red), and DAPI (blue) **(A2)** Same as **A1**, but for a retinal from the OEC-injected group, showing grafted OECs (green, EGFP), and a reduced number of TMEM119-positive cells (white arrows). **(B)** mRNA expression level of TMEM119 in the OEC-group, relative to expression in PBS group (*n* = 3 per bar). **(C)** Example western blot of TMEM119 protein levels. **(D)** Quantitative group data of TMEM119 protein levels in OEC-group, normalized to β-actin expression levels, and compared to PBS-group retinas (*n* = 3 per bar). **(E–H)** Same as **A–D**, but for Iba1. ^*^p < 0.05, ^∗∗^*p* < 0.01; Scale bars: 50 μm.

Firstly, and in agreement with the previous *in vitro* results, immunohistochemistry of retinas from the *in vivo* model demonstrated that the number of TMEM119-positive cells (activated resident microglia; *white arrows*, Figure 7A) was reduced in RCS rats treated with OECs compared with the control (PBS-injected) group. Secondly, we collected homogenized retinal tissue and used RT-PCR and WB confirmed that, TMEM119 gene expression at 4 weeks in the OEC-group was 0.56 ± 0.146 of that in the control group (Figure 7B; *n* = 3 per group; *p* < 0.05 vs. PBS control), and TMEM119 protein levels were 0.56 ± 0.13 of that of the control group, both were significantly lower than PBS group (*n* = 3 per group; *p* < 0.05; Figures 7C,D). However, the number of Iba1-positive cells in the retina did not seem decreased after OEC transplantation (Figure 7E). Even Iba1 mRNA levels (Figure 7F) and protein levels (Figures 7G,H) were comparable between OEC and control groups (*p* > 0.05 for each).

As we know, microglia are the primary immune cells in the retina, which share many phenotypic and functional properties with macrophages. We have already confirmed OECs have effect on activated microglia *in vitro* and *in vivo*, why the common marker of activated microglia and infiltrated macrophage Iba1 keep stable? We additionally used double-staining of NGFRp75 and Iba1 in purified OECs and in RCS rat after transplantation (Supplementary Figure S4). The result showed OECs not only express Iba1 *in vitro*, which merged with it’s special marker NGFRp75 (Supplementary Figure S2A), but also the Iba1 positive cells *in vivo* stain positive with GFP-OEC after transplantation (Supplementary Figure S4B). This circumstantial result indicated that transplanted OECs may also affect Iba1 expression *in vivo*, but due to OEC express Iba1 itself, the Iba1 expression amount keep stable in whole retinal detection.

**FIGURE 8:**
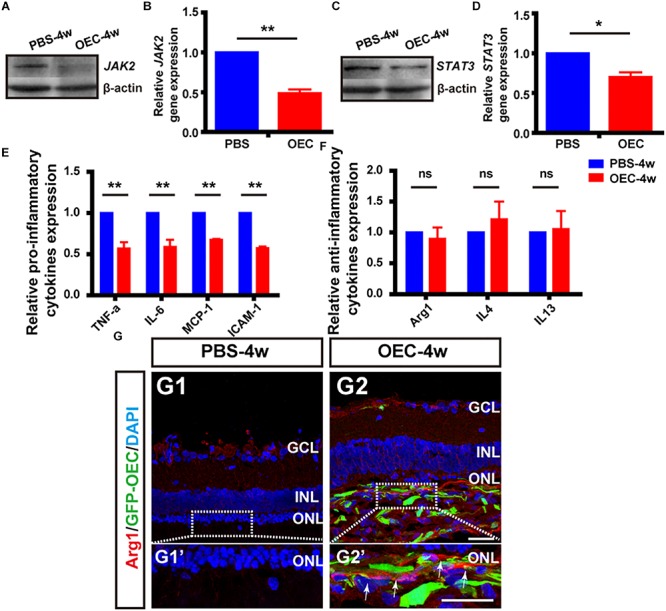
OECs inhibit the JAK2/STAT3 pathway and change the expression level of pro-inflammatory factors *in vivo*. **(A,B)** Example WB and group data showing effect on JAK2 expression of OEC transplantation, 4 weeks post-injection. **(C,D)** As per A, B, but for STAT3 expression. **(E)** mRNA expression levels of pro-inflammatory factors: TNF-α, IL-6, ICAM-1 and MCP-1 (*n* = 3 per bar, *p* < 0.05 for both vs. PBS-group control). **(F)** mRNA expression levels of anti-inflammatory factors: IL-4, IL-13, and Arg1 (*n* = 3 per bar, *p* > 0.05 for both vs. PBS-group control). **(G)** Immunohistology image, labeled with Arg1 (*red*) and DAPI (*blue*), of a retina from the PBS-group **(G1)** and OEC-group **(G2)**, indicating increased Arg1-positive cell numbers in the transplantation area (*white arrows*). ^*^p < 0.05, ^∗∗^*p* < 0.01; Scale bars: 50 μm.

Having shown *in vitro* that OECs changed the expression level of pro- and anti-inflammatory factors of microglial through the JAK2/STAT3 pathway, we wanted to investigate it *in vivo.* We performed WB analysis of protein lysates from retinas from the OEC group at 4 weeks post-transplantation. This showed that JAK2 expression was reduced to 0.49 ± 0.05 of control group levels (*n* = 3 per group; *p* < 0.01; Figures 8A,B) and STAT3 was reduced to 0.70 ± 0.06-fold of control group levels (*n* = 3 per group; *p* < 0.05; Figures 8C,D). These results indicate that OEC treatment reduced the activation of the JAK2/STAT3 pathway in the degenerative RCS retina. RT-PCR analysis showed in the OEC group, there was a significant reduction in mRNA expression of the pro-inflammatory cytokines compared to control (*n* = 3 per bar; *p* < 0.01 for each vs. control) (Figure 8E). In contrast, we found that anti-inflammatory factors were not affected by OEC transplantation *in vivo* (Figure 8F; *n* = 3 per bar, *p* > 0.05 for each vs. control). However, it did appear from immunostaining that there were a larger number of Arg1-positive cells around the transplantation site in the OEC group, compared to the PBS group (Figure 8G). Maybe the differences between RT-PCR and immunostaining indicated the expression level of anti-inflammatory factors were so low and just in the transplantation area that could not be detected in whole retina tissue.

### OECs Delay Retinal Degeneration in RCS Rats

In previous studies, we have shown that transplanted OECs protect photoreceptors by releasing multiple neurotrophic factors, phagocytosing out segment, inhibiting Müller cell gliosis, and suppressing retinal oxidative stress reactions in rat models of retinal degeneration (Huo et al., 2011; Xie et al., 2017; Xue et al., 2017). Regardless of the model of retinal degeneration, we saw peak visual function improvement at 4 weeks post OEC transplantation. In the current study in RCS rats, we have shown a reduction in classically microglial activation and pro-inflammatory cytokine release at the same time point post OEC transplantation, and we therefore also tested visual function and observed retinal anatomy this key time point.

**FIGURE 9:**
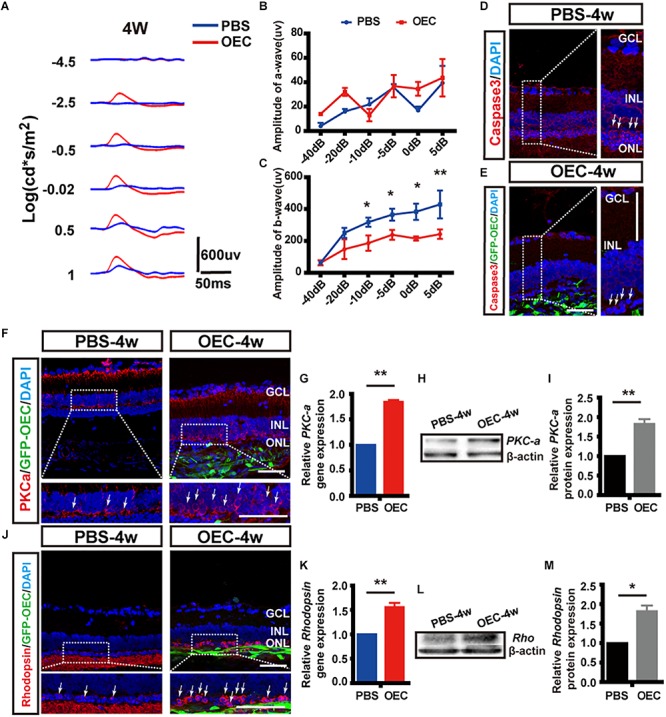
Subretinal OEC transplantation delays functional and anatomical retinal degeneration in RCS rats. **(A)** Representative ERG waveforms of a rat at 4 weeks post-OEC transplantation (red trace), or PBS control injection (blue trace). **(B)** ERG a-wave and **(C)** ERG b-wave amplitude at 4-weeks post OEC transplantation (vs. PBS control injection, *n* = 6). **(D,E)** Immunohistology images, labeled for caspase-3 (red), GFP (green, OECs) and DAPI (blue) in the retinas of the PBS (top), and OEC-treated (bottom) groups. White arrows indicate caspase-3 positive cells. **(F)** Immunohistology images, labeled for PKC-α (red) GFP (green, OECs) and DAPI (blue) in the retinas of the PBS (left) and OEC-treated (right) groups. White arrows indicate PKC-α positive cells. **(G)** Immunohistology images, labeled for Rhodopsin (red) GFP (green, OECs) and DAPI (blue) in the retinas of the PBS (left) and OEC-treated (right) groups. White arrows indicate Rhodopsin positive cells. **(H,I)** The mRNA level of PKC-α **(H)** and Rhodopsin **(I)** in the OEC-group at 4 weeks post-transplantation, expressed relative to the PBS control group. **(J,K)** Example western blot of PKC-α **(J)** and Rhodopsin **(K)** at 4 weeks post-treatment. **(L,M)** Quantification of protein expression level of PKC-α protein **(L)** and Rhodopsin **(M)** at 4 weeks post-treatment, measured relative to β-actin, and expressed relative to the PBS control group. ^*^p < 0.05, ^∗∗^*p* < 0.01. ONL, outer nuclear layer; INL, inner nuclear layer; GCL, ganglion cell layer. Scale bars: 100 μm in **A**; 50 μm in **B,H,I,J,N**; 20 μm in each enlargement.

To determine visual function, we measured the amplitudes of the a-wave and b-wave from the ERG (Figure 9A). These are electrical features that are associated with the function of the outer and inner retina, respectively. B-wave amplitude was significantly larger in OEC-treated eyes than in PBS-treated controls (*p* < 0.05; Figure 9C). This suggested an improvement in inner-retinal function following OEC transplantation. However, there was no significant difference in a-wave amplitude between the transplant group and control group (*p* > 0.05; Figure 9B). As an additional negative control, we transplanted ONFs using the same procedure as OECs, and found no improvement in b-wave amplitude at 2, 3, or 4 weeks post-transplantation (Supplementary Figure S5).

We next studied changes in the anatomical structure of the retina. During retinal degeneration, the number of photoreceptors in the ONL is reduced and the expression of Caspase-3 is increased (Gu et al., 2017). Using histological methods, we found that OEC reduced the expression of Caspase-3 in transplantation area, and there was noticeably increased preservation of photoreceptor numbers in the ONL (white arrows, Figures 9D,E). This suggested that the apoptosis of photoreceptors in RCS rats was reduced by OEC transplantation.

Immunohistochemistry showed that the expression of both PKC-α (Figure 9F), a marker of ON-bipolar cells (which contribute to the b-wave), and Rhodopsin (Figure 9G), a marker of photoreceptors (which contribute to the a-wave), was increased at 4 weeks post-OEC transplantation, and compared to PBS controls.

Finally, RT-PCR and WB were used to quantify gene and protein expression. The gene expression ratio of both PKC-α and Rhodopsin was significantly increased in the OEC-group compared with the PBS-group (PKC-α: 1.84 ± 0.04 fold, Figure 9H, *n* = 3 per group; Rhodopsin: 1.56 ± 0.09 fold, Figure 9I, *n* = 3 per group; *p* < 0.01 for each vs. PBS control). WB (Figures 9J,K) also showed up-regulation of PKC-α and Rhodopsin protein following OEC transplantation (PKC-α: 1.82 ± 0.12 fold, Figure 9L, *n* = 3 per group; Rhodopsin: 1.81 ± 0.15 fold, Figure 9M, *n* = 3 per group; *p* < 0.05 for both vs. PBS control). These results collectively demonstrate that OEC transplantation can protect photoreceptors and ON-bipolar cells, and preserve the ERG b-wave in the RCS retina.

## Discussion

In the present study, we have shown how OECs can affect LPS-induced microglial activation profiles *in vitro*, how OEC transplantation *in vivo* can moderate the inflammatory microenvironment, protect photoreceptor and improve visual function, in chronic retinal degeneration animal model. Our results demonstrate that during RCS rats’ retinal degeneration, classically activated immune cells up-regulated JAK2/STAT3 pathway and produce persistent pro-inflammatory factors, but only transient anti-inflammatory factors in early stage. *In vitro* experiments showed that OECs exert immunomodulatory effects through a change of expression level of inflammatory factors in LPS-induced microglia activation: away from M(IL-6)-type (TNF-α, IL-6, MCP-1 and ICAM-1), toward M(Arg1)-type (Arg1, IL-4, IL-13), via a mechanism that is dependent on the JAK2/STAT3 pathway. OEC transplantation was able to improve the inflammatory microenvironment of RCS rats *in vivo*: indicated by a reduction in microglial/macrophage activation, down-regulated JAK2/STAT3 pathway and pro-inflammatory factors expression level. Finally, OEC transplantation helps preserve visual function and delay retinal neuron degeneration, partly via these effects on the microglia/macrophage-mediated inflammatory microenvironment.

Resident microglia have been reported to be rapidly activated and migrate toward the inflamed lesion under pathological conditions in the retina (Akhtar-Schäfer et al., 2018; Szepesi et al., 2018). In RCS rats with *Mertk* gene mutation, BRB disruption results in the recruitment of blood-borne macrophages to help phagocytosis the apoptotic photoreceptors (Fernández-Sánchez et al., 2018). In our study, TMEM119 was applied to distinguish resident microglia and infiltrating macrophages. Consistent with previous studies (Greenhalgh et al., 2018; Li et al., 2018), in the normal retina, we observed resident microglia with a quiescent, ramified morphology, with low Iba1, and TMEM119 staining. But in RCS rats, a large number of TMEM119-positive cells were only located in the ONL, whilst Iba1-positive cells located from the GCL to the subretinal space. Cells positive for both types of staining increased from the early stages (P30), and reached peak expression at around P60. As we know, Iba1 is the most common marker for activated microglia and macrophages. TMEM119 is a newly discovered and stable marker with unknown function for most or all mouse and human microglia, and it has been demonstrated that bone marrow-derived macrophages in the adult CNS do not express TMEM119 (Bennett et al., 2016). However, no one reported the expression manner of TMEM119 in rat’s retina. Therefore, the present study revealed that TMEM119^+^ cells located in ONL during retinal degeneration may stand for a subpopulation of resident microglia which associated with photoreceptor apoptosis, along with the Iba1^+^ microglia/infiltrating macrophages, play a crucial role in releasing of pro-inflammatory cytokines, and amplify the neurodegenerative disease process.

Emerging evidence suggests that there is a dynamic change of microglia/macrophage during the process of retinal degeneration. Jiao et al. (2015) reported the cytokines released by retinal microglia/macrophage changed from pro- to anti-inflammation-type following acute light injury from 24 h to 7 days. In rd1 mice (a rapid-onset model of RP) with rapid rods degenerative process, the activated microglia adopted M(IL-6)-dominent phenotype and lack of M(Arg1)-dominent phenotype (Zhou et al., 2017). In the P23H mice (a chronic RP model), retinal neuroinflammation persists throughout the mice life span, even after photoreceptor depletion (Noailles et al., 2016). In the present study, we found evidence of a prominent and persistent M(IL-6) phenotype and a transient M(Arg1) phenotype in a RCS rat model of RP. Pro-inflammatory cytokines expression was increased in early life time, and reached a peak (hundreds of times higher than normal rat retinas) around P60. Anti-inflammatory cytokines expression was also increased many-fold at P30, but by P60, fell below control levels. These results suggest that some anti-inflammatory cytokines are activated in the degenerative retina to enhance debris phagocytosis, release protective/trophic factors, and support regeneration in the early stages. However, as degeneration continues, pro-inflammatory cytokines are robustly releasing by M(IL-6)-type microglia/macrophages, causing expanding tissue damage, and worsen disease. Interestingly, the microglia/macrophage activation pattern found in this study, with an early peak in anti-inflammatory factors expression, is in contrast to the more commonly observed pro- to anti-inflammatory cytokines shift described in the injury in CNS, including in spinal cord injury (SCI) (Kigerl et al., 2009), stroke (Hu et al., 2012), and traumatic brain injury (Wang et al., 2013).

Two recent studies have demonstrated that OECs can change the microglia/macrophage polarization toward M(Arg1) in a SCI model, and this is accompanied by an inhibition of local inflammatory responses (Khankan et al., 2016; Zhang et al., 2017), but they didn’t mention the possible mechanism. To investigate the specific immunomodulatory effect of OECs on microglia in retina, we used an *in vitro* Transwell co-culture system and an *in vivo* OEC transplantation model. *In vitro*, we found that OECs not only reduced Iba1 and TMEM119 expression induced by LPS which stand for microglia cell line activation, but also changed the cytokines expression level of BV2 cells from the destructive M(IL-6) phenotype toward the neuroprotective and tissue-reparative M(Arg1) phenotype. Because of the complex microenvironment, the result of *in vivo* studies were not all the same as *in vitro* ones. In RCS rats, we confirmed a reduction of TMEM119^+^ resident microglial activation and a stable expression of Iba1. As we reported before, OECs, like microglia, and have the function of phagocytosis (Li et al., 2017). Some anatomy studies also suggested OECs are the primary immune cells in olfactory nervous system (Gladwin and Choi, 2015; Barton et al., 2017), but there is no reported correlation between Iba1 and OEC in past studies. From our study, immunohistochemistry demonstrates OECs co-expressed Iba1 and its specific marker NGFRp75. Presumably although the transplanted OECs suppressed the Iba1 level expressed by both resident microglia and infiltrated macrophages in transplanted area, due to the OEC itself also expressed Iba1, the whole detection level in post-transplanted retina did not vary significantly. RT-PCR result showed a decrease in pro-inflammatory cytokines and a stable in anti-inflammatory cytokines expression from whole retinal detection. Although the mRNA level of anti-inflammatory cytokines did not show significant change, the immunohistochemistry showed that Arg1-positive cells were notably increased surrounding transplantation area. We suspected OEC may increase anti-inflammatory cytokines in the transplantation area, but as the amount is limited, it couldn’t be detected in whole retinal tissue.

Mechanistically, the most critical question to address is how OECs regulate microglial activation and the M(IL-6)/M(Arg1) phenotype switch. Among numerous pathways, the JAK/STAT pathway is thought to be key regulator. It is reported that IFN-γ induced STAT1 activation leads to an increase in production of pro-inflammatory cytokines, and programs microglia/macrophages to the M(IL-6) phenotype (Qin et al., 2012). In contrast, STAT6 activation contributes to anti-inflammatory M(Arg1) phenotype and the release of neurotrophic factors, Arg1, IL-4, and IL-13. STAT3 involve both Arg1-stimulated M2 polarization and IL-6-stimulated M1 polarization (Martinez and Gordon, 2014). In our research, i*n vivo* experiments demonstrated that the JAK2/STAT3 pathway became activated during degenerative period which was the same as research on retinal degeneration has shown that JAK2 and STAT3 proteins are involved in photoreceptor apoptosis, especially in rd1 mice model (Samardzija et al., 2006; Lange et al., 2010). After OEC co-cultured or transplanted, both JAK2 and STAT3 protein expression was strongly reduced. By using a specific chemical antagonist of JAK2, AG490 to pretreat OEC, we were able to demonstrate that the mechanism by which OECs appear to reduce retinal microglial activation and inflammation is JAK2-dependent. Recent studies about macrophage polarization have established strong potential in suppressing of cytokine signaling (SOCS) proteins. SOCS proteins, especially SOCS3, not only suppresses JAK tyrosine kinase activity and negatively regulate JAK/STAT pathway, but also inhibits gp130-related cytokine receptors and abrogates IL-6-induced pro-inflammatory effects (Carow and Rottenberg, 2014). In macrophages, SOCS3 inhibits STAT3 activity preserving the natural cytotoxicity of M1 and develop characteristics of M2a activated cells (Liu et al., 2008). Qin et al. reported astrocytes expressed SOCS1 and SOCS3 (Qin et al., 2008) while Girolami et al. (2010) found SOCS3 expression is restricted mainly to Schwann cells in peripheral nerve injury. Consistent with both astrocytes and Schwann cells, SOCS3 immunoreactivity was observed in OECs. The mRNA expression of SOCS3 in OECs was enhanced significantly from 12 to 48 h after co-cultured with LPS induced BV2 cells. As during retinal degeneration, the apoptosis of photoreceptors and glia cell produce a large number of IL-6, which band to gp130, and form a complex to activate the kinase function of JAK2. JAK2 then phosphorylate STAT3, two phosphorylated STAT3 translocate into the cell nucleus, and promote pro-inflammatory factors gene transcription (Yin et al., 2015). We speculated as the OECs high expressing SOCS3, after co-cultured or transplanted, the IL-6 was regulated, and the cascade JAK/STAT pathway was inhibited. Suppression of the JAK/STAT pathway result in a switch of the proinflammatory phenotype to the anti-inflammatory M2 phenotype, as M2-related genes, such as IL-4 and Arg1, were altered significantly *in vitro*.

Finally, we found substantial improvement in visual function and anatomical retinal structure following OEC transplantation in RCS rats. OECs led to nearly a twofold increase in the amplitude of the ERG b-wave, a significant inhibition of Caspase-3 expression, a up-regulated gene and protein expression of PKC-α and Rhodopsin protein which stand for ON-bipolar cells and photoreceptors. Similarly, OECs have been found to modulate the host immune response, and promotes preservation of neurons and axons in a rat model of SCI (Khankan et al., 2016). In addition, transplanted OEC promote the macrophage shift from M(INF-γ) to M(IL-4) and significantly improve motor function after SCI (Zhang et al., 2017). Therefore, combined with the previous research, we conducted the OEC injection modulate microenvironment through secrete neurotrophic factors, inhibit Müller cell gliosis, anti-oxidant, and immunomodulatory mechanisms in retinal degenerative models (Huo et al., 2011, 2012; Xie et al., 2017; Xue et al., 2017) to protect photoreceptors and inner neurons. Finally, in this study, we demonstrated that OECs have effect on activated microglia polarization from M(IL-6) to M(Arg1) through JAK2/STAT3 pathway. In addition, alleviation of activated resident microglia and reduction of pro-inflammatory microenvironment by OEC transplantation contributed to substantial visual function and structure improvement.

## Data Availability

The raw data supporting the conclusions of this manuscript will be made available by the authors, without undue reservation, to any qualified researcher.

## Ethics Statement

All surgical procedures and post-operative care were conducted in accordance with protocols approved by the TMMU Institutional Animal Care and Use Committee.

## Author Contributions

JX conceived and designed the study, collected and assembled the data, carried out the data analysis, interpreted the data, and wrote the manuscript. YL, JD, YH, DS, and CD collected and assembled the data. HX conceived and designed the study, carried out the data analysis, interpreted the data, and wrote and approved the final version of the manuscript. ZY conceived and designed the study, was responsible for the financial support, carried out the data analysis, interpreted the data, and approved the final version of the manuscript.

## Conflict of Interest Statement

The authors declare that the research was conducted in the absence of any commercial or financial relationships that could be construed as a potential conflict of interest.
