# Supplementary material for: Olfactory Ensheathing Cells Grafted Into the Retina of RCS Rats Suppress Inflammation by Down-Regulating the JAK/STAT Pathway
Source: Front Cell Neurosci. 2019 Jul 25;13:341. doi: 10.3389/fncel.2019.00341 (PMC6670006; doi:10.3389/fncel.2019.00341)
Supplement: Supplementary file 1 [file Data_Sheet_1.pdf]

Olfactory ensheathing cells grafted into the retina of RCS rats suppress inflammation by down-regulating the JAK/STAT pathway

Jing Xie[1,2], Yijian Li[1,2], Jiaman Dai[1,2], Yan He[1,2], Dayu Sun[1,2], Chao Dai[1,2], Haiwei Xu[1,2#], and Zheng Qin Yin[1,2#]

1. Southwest Hospital/Southwest Eye Hospital, Third Military Medical University, Chongqing, 400038, P.R. China

2. Key Lab of Visual Damage, Regeneration and Restoration of Chongqing, Chongqing, 400038, P.R. China

#, Corresponding authors

E-mail: qinzyin@aliyun.com (ZQY); haiweixu2001@163.com (HWX)

Table 1. Primary antibodies used.

Antibody	Manufacturer's catalog or lot number	Dilution
Mouse anti-NGFRp75	Santa Cruz, sc-271708	1: 50
Rabbit anti-Iba1	Wako, 019-19741(IFC)	1: 500
	016-20001(WB)	1:1000
Rabbit anti-TMEM119	Abcam, ab185333 (IHC)	1: 50
	Santa Cruz, sc-244341 (WB)	1: 500
Mouse anti-PKCα	Santa Cruz, Santa Cruz, sc-8393	1: 500
Mouse anti-Rhodopsin	Abcam, ab5417	1: 1000
Rabbit anti-S-100β	Abcam, ab868	1: 200
Rabbit anti-Caspase-3	Abcam, ab13847	1: 200
Mouse anti-β-actin	Cell Signal Technology, 3700	1:2000
Rabbit anti-JAK2	Cell Signal Technology, 3230	1: 1000
Rabbit anti-STAT3	Cell Signal Technology, 12640	1: 1000
Rabbit anti-SOCS3	Abcam, ab16030	1:100
Rabbit anti-pJAK2	Cell Signal Technology, 3776	1: 1000
Rabbit anti-pSTAT3	Cell Signal Technology, 9145	1: 1000

Table 2. PCR primer sequences (Rat) used to detect pro- and anti-inflammatory cytokines in RCS rats' retina

Genes (Rat)	Forward primer	Reverse primer
GAPDH	AAGGTCGGTGTGAACGGATT	TGAACTTGCCGTGGGTAGAG
TNF-α	CTCAAGCCCTGGTATGAGCC	GGCTGGGTAGAGAACGGATG
IL-6	TCCTACCCCAACTTCCAATGC	TAGCACACTAGGTTTGCCGAG
MCP-1	GCTGTAGTATTTGTCACCAAGCTCAA	GTACTTCTGGACCCATTCCTTATTG
ICAM-1	AGTGCTGTACCATGATCAGAATACCT	TAAATGGACGCCACGATCAC
Arg1	CCTGAAGGAACTGAAAGGAAAGTT	GCAAGCCGAT GTACACGATGT
IL-4	ACCCTGTTCTGCTTTCTC	GTTCTCCGTGGTGTTCCT
IL-13	AATCCCTGACCAACATCT	ATAAACTGGGTACTTCG
Iba1	CGAATGCTGGAGAAACTTGG	GTTGGCTTCTGGTGTTCTTTG
TMEM119	GCTACGCTTTCTTCACGTTGC	AACCAATCAGGAAGTGGGGT
PKC-α	TTTCTTCCCCCACCCAATCC	AGGGTCCAAGTCTCTTTGTTTCC
Rhodopsin	AACCTTGAGGGCTTCTTTGCCA	AAGTTGCTCATGGGCTTGGAGA
SOCS3	TTCTTTACCACCGACGGAAC	CACGTTGGAGGAGAGAGGTC

Table 3. PCR primer sequences (Mice) used to detect pro- and anti-inflammatary cytokines in BV2 cells

Genes (Mice)	Forward primer	Reverse primer
GAPDH	CAGCAACTCCCACTCTTCCAC	TGGTCCAGGGTTTCTTACTC
TNF-α	TGTGCTCAGAGCTTTCAACAA	CTTGATGGTGGTGCATGAGA
IL-6	TAGTCCTTCCTACCCCAATTTCC	TTGGTCCTTAGCCACTCCTTC
Arg1	ACAAGACAGGGCTCCTTTCAG	GGCTTATGGTTACCCTCCCG
IL-4	ATCCATTTGCATGATGCTCT	GAGCTGCAGAGACTCTTTCG
Iba1	GGATTTGCAGGGAGGAAAAG	TGGGATCATCGAGGAATTG
TMEM119	GTGTCTAACAGGCCCCAGAA	AGCCACGTGGTATCAAGGAG
