# Supplementary material for: Olfactory Ensheathing Cells Grafted Into the Retina of RCS Rats Suppress Inflammation by Down-Regulating the JAK/STAT Pathway
Source: Front Cell Neurosci. 2019 Jul 25;13:341. doi: 10.3389/fncel.2019.00341 (PMC6670006; doi:10.3389/fncel.2019.00341)
Supplement: Supplementary file 2 [file Data_Sheet_2.pdf]

Olfactory ensheathing cells grafted into the retina of RCS rats suppress inflammation by down-regulating the JAK/STAT pathway

Jing Xie[1,2], Yijian Li[1,2], Jiaman Dai[1,2], Yan He[1,2], Dayu Sun[1,2], Chao Dai[1,2], Haiwei Xu[1,2#], and Zheng Qin Yin[1,2#]

1. Southwest Hospital/Southwest Eye Hospital, Third Military Medical University, Chongqing, 400038, P.R. China

2. Key Lab of Visual Damage, Regeneration and Restoration of Chongqing, Chongqing, 400038, P.R. China

#, Corresponding authors

E-mail: qinzyin@aliyun.com (ZQY); haiweixu2001@163.com (HWX)

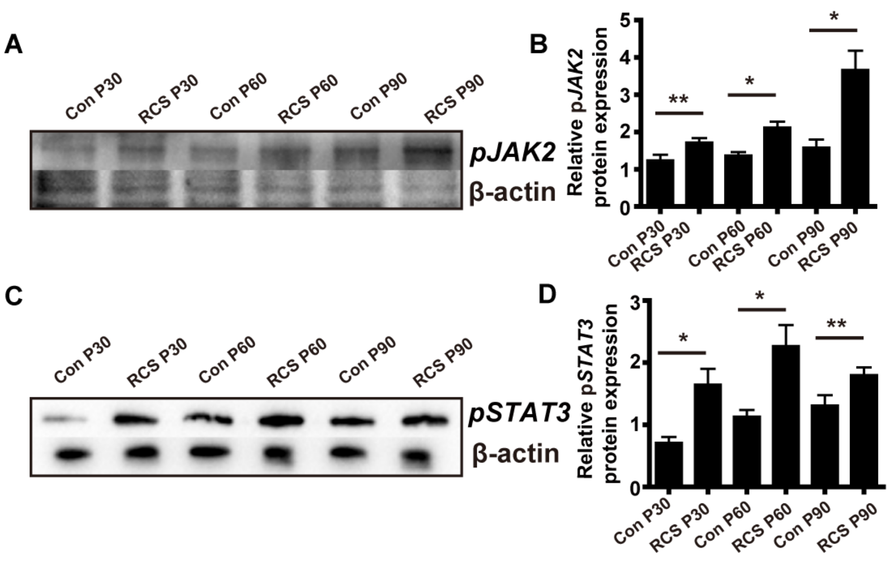

Figure S1. pJAK2/pSTAT3 activation during retinal degeneration.

(A) WB results for protein levels of pJAK2 in retinas from RCS rats and control rats of different ages. (B) Group data showing pJAK2 expression by western blot in RCS rats, relative to the β-actin. (C, D) The same as A, B, but for pSTAT3. (n = 3 per bar). **p* < 0.05, ***p* < 0.01.

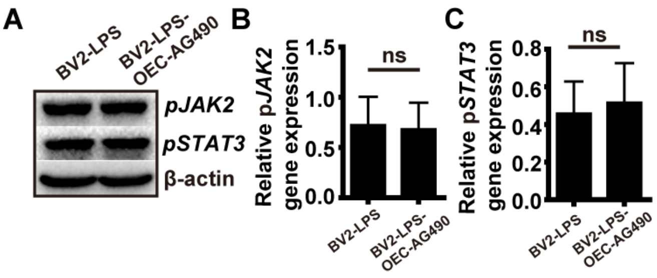

Figure S2. The change of pJAK2/pSTAT3 in BV2-LPS and BV2-LPS-OEC-AG490 group.

(A) WB results for protein levels of pJAK2 in BV2-LPS and BV2-LPS-OEC-AG490 group. (B, C) Group data showing pJAK2 and Pstat3 expression by western blot in BV2-LPS and BV2-LPS-OEC-AG490 group, relative to the β-actin. (n = 3 per bar). **p* < 0.05, ***p* < 0.01.

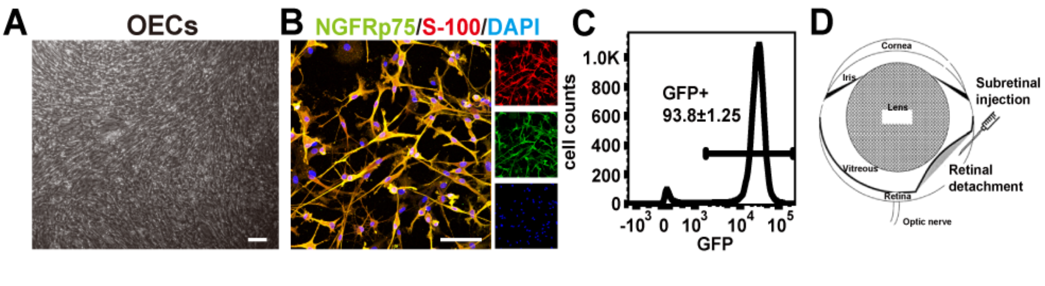

Figure. S3. *OEC purification, identification, transfection with lentivirus-EGFP and subretinal transplantation of labelled OECs.*

(**A**) Morphology of OECs after primary culture and purification. (**B**) Merged image showing immunostaining to identify OECs. Inserts show separate color channels: NGFRp75 (*green*); S-100β (*red*); DAPI (*blue*). (**C**) Flow cytometry data showing the percentage of GFP-positive OECs. (**D**) Schematic illustration of subretinal cell transplantation in the rat eye.

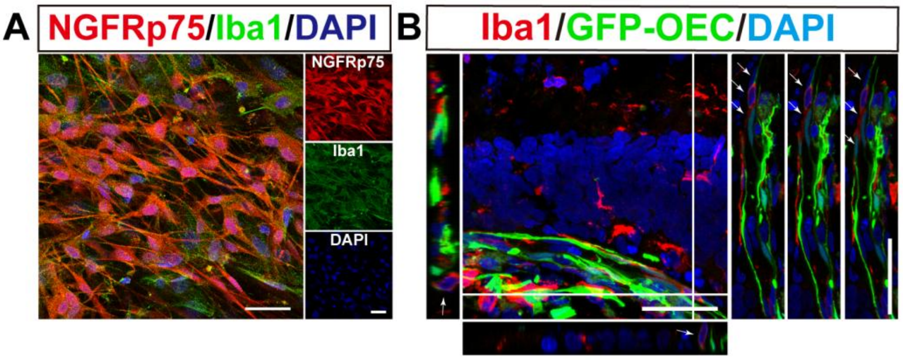

Figure. S4. *OECs express Iba1 in vitro and in vivo.*

(A) Merged image showing immunostaining of purified OECs in vitro. Inserts show separate color channels: NGFRp75 (red), Iba1 (green) and DAPI (blue). Scale bar: 50 μm (**B**) Orthogonal view of an in vivo GFP-labeled (green) OEC which also stains positive for Iba1 (red). Right inserts show three different layers of Double-stained cell. The double-stained cell is marked with white arrows. Scale bar: 25 μm.3.6. OEC transplantation down-regulates the JAK/STAT pathway and changed the expression level of pro- inflammatory factors in RCS rat retinas.

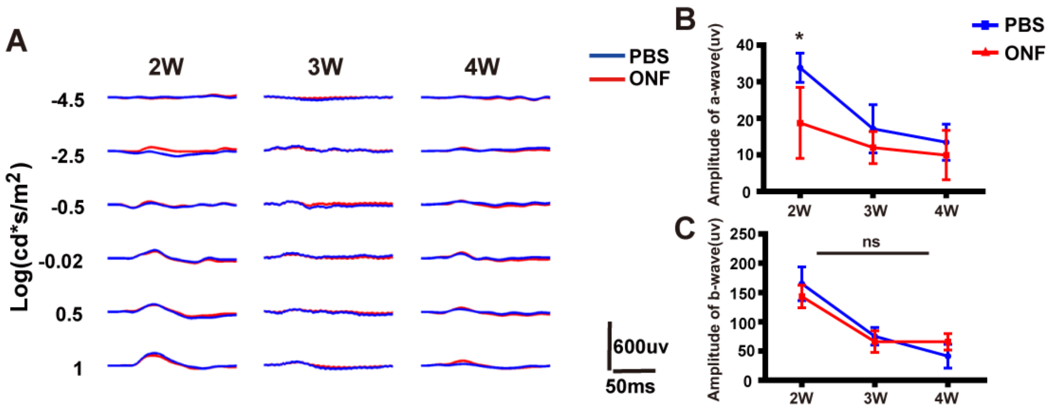

Figure. S5. *Subretinal ONF transplantation has no effect on the ERG in RCS rats.*

(**A**) Representative ERG waveforms of rats from 2–4 weeks post-ONF transplantation (*red trace*), or PBS control injection (*blue trace*). (**B**) ERG a-wave and (**C**) ERG b-wave amplitude at 2-4 weeks post OEC transplantation (versus PBS control injection).
